# Genetic polymorphism of matrix metalloproteinase 9 and susceptibility to chronic obstructive pulmonary disease: A meta-analysis

**DOI:** 10.5937/jomb0-34155

**Published:** 2022-07-29

**Authors:** Xiaoping Yang, Yuanyuan Yu, Yong Wang, Wen Jiang, Wenqing Jiang, Bin Yin

**Affiliations:** 1 Qingdao Hospital of Traditional Chinese Medicine (Haici Hospital), Department 2 of Respiratory and Critical Care (Lung disease) Center, Qingdao, China; 2 Qingdao Hospital of Traditional Chinese Medicine (Haici Hospital), Department of Anesthesiology, Qingdao, China

**Keywords:** MMP9, polymorphism, COPD, meta-analysis, MMP9, polimorfizam, HOBP, meta-analiza

## Abstract

**Background:**

To systematically analyze the influence of genetic polymorphisms of matrix metalloproteinase 9 (MMP9) on susceptibility to chronic obstructive pulmonary disease (COPD).

**Methods:**

Relevant literatures reporting MMP9 and susceptibility to COPD in PubMed, Web of Science, VIP, Wanfang and CNKI databases were searched using the key words "matrix metalloproteinases 9/MMP9, COPD/chronic obstructive pulmonary disease". Data of eligible literatures were extracted and analyzed for the odds ratio (OR) and corresponding 95% CI.

**Results:**

A total of 16 independent studies reporting MMP9-1562C/T and COPD patients were enrolled and analyzed. None of the genetic models revealed the relationship between MMP9-1562C/T and susceptibility to COPD. Subgroup analyses identified lower risk of COPD in Chinese population carrying the TT genotype for theMMP9 rs3918242 relative to those carrying CT and CC genotypes (P=0.03, OR=0.67, 95% CI=0.46-0.97).

**Conclusions:**

Chinese population carrying the TT genotype for the MMP-9 rs3918242 present lower susceptibility to COPD relative to those carrying CT and CC genotypes.

## Abstract (sr)

**Uvod:**

Sistematska analiza uticaja genetskih polimorfizama matriks metaloproteinaze 9 (MMP9) na osetljivost hronične opstruktivne bolesti pluća (HOBP).

**Metode:**

Relevantna literatura koja izveštava o MMP9 i podložnosti HOBP u bazama podataka PubMed, Web of Scince, VIP, Wanfang i CNKI pretraživana je korišćenjem ključnih reči "matriks metaloproteinaze 9/MMP9, COPD/hronična opstruktivna bolest pluća". Podaci iz kvalifikovane literature su ekstrahovani i analizirani za odnos šanse (OR) i odgovarajući 95% CI.

**Rezultati:**

Ukupno je uključeno i analizirano 16 nezavisnih studija koje su izveštavale o pacijentima sa MMP9-1562C/T i HOBP. Nijedan od genetskih modela nije otkrio vezu između MMP9-1562C/T i osetljivosti na HOBP. Analize podgrupa identifikovale su niži rizik od HOBP kod kineske populacije koja nosi TT genotip za MMP-9 rs3918242 u odnosu na one koji nose CT i CC genotipove (P=0,03, OR=0,67, 95% CI=0,46-0,97).

**Zaključak:**

Kineska populacija koja nosi TT genotip za MMP9 rs3918242 predstavlja manju osetljivost na HOBP u odnosu na one sa CT i CC genotipovima.

## Introduction

Chronic obstructive pulmonary disease (COPD) is a worldwide disease affecting approximately 3 million people. It is estimated that COPD will be the third leading cause of death by 2020 [1]. As a chronic airway inflammatory disease, COPD is characterized by incomplete reversible airflow limitation, inflammatory cell infiltration, excessive mucus secretion, and airway remodeling [2]. The precise molecular mechanism underlying the pathogenesis of COPD remains unclear. At present, it is generally believed that several risk factors are directly related to the pathogenesis of COPD, including host and environmental factors [3]. Among environmental factors, smoking, exposure to chemicals, indoor and outdoor air pollution are risk factors for COPD [4]. Host factors of COPD include antitrypsin-1, excessive deposition of extracellular matrix (ECM), corticosteroids, inflammatory stimuli, and metabolic imbalances [5] [6].

Matrix metalloproteinases (MMPs) are members of the metformin group and they are capable of degrading ECMs and regulating extracellular signaling networks [7]. MMPs are important in COPD. They degrade matrix proteins (elastin, collagen) during the disease progression [8]. In the past decade, abundant researches have been conducted to analyze the relationship between single nucleotide polymorphisms (SNPs) of MMPs and COPD risk in some populations [9] [10] [11] [12]. However, the conclusions were controversial. Some reports demonstrated the certain influence of MMPs on the occurrence of COPD [13] [14] [15] [16] [17] [18], while others did not [9] [12] [19] [20]. These conflicting findings may be explained by limited sample size, false positive results, and publication bias. In this paper, we performed a comprehensive meta-analysis to assess the influence of MMP polymorphisms on COPD.

## Materials and methods

### Search strategy of literatures

Relevant literatures reporting the relationship between polymorphisms of MMP9-1562C/T and susceptibility to COPD in PubMed, Web of Science, VIP, Wanfang and CNKI databases were searched using the key words »matrix metalloproteinases 9/MMP9, COPD/chronic obstructive pulmonary disease«. There were no limitations on published languages. Citations in each literature were manually reviewed.

### Inclusive and exclusive criteria

Inclusive criteria were as follows:1) Case-control studies conducted in humans; 2) Literatures published complete data or raw data that could calculate the genotype distribution; 3) COPD patients underwent diagnosis of pulmonary function index; 4) Literatures were conducted on the influence of polymorphisms of MMP9-1562C/T on susceptibility to COPD.

Exclusive criteria were as follows: 1) Repeated literatures; 2) Literatures lacked valid raw data; 3) Reviews, comments, animal experiments, researches on mechanism and case reports;4) The latest studies or those with a larger sample size were selected if data overlapping; 5) Unpublished data.

**Figure 1:**
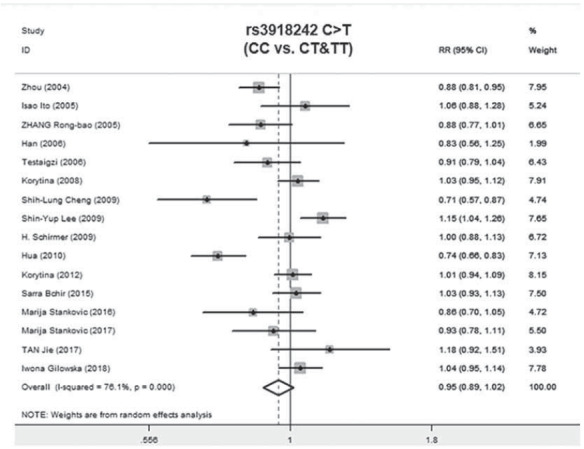
Flow diagram of the publication selection process

Flow diagram of literature searching was depicted in Figure 1.

### Data extraction

Data were independently extracted and analyzed by two researchers, and the third one was responsible for solving any disagreement. Extracted data included: 1) Baseline data of literatures, including publication origin, first author, year or publication, and etc.; 2) Basic characteristics of subjects, including sample size, research country, genotype number and distribution, HWE in control group and etc.

### Statistical analysis

Heterogeneity test was conducted by calculating odds ratio (OR) and the corresponding 95% CI with the *I* *^2^* test and the Q test. The pooled OR in studies lacking the heterogeneity was calculated by the fixeffects model. Otherwise, a random-effects model was used. Sensitivity analysis was performed by removing one study each time and analyzing the remaining in a combination way. The HWE of control genotype distribution was evaluated using the x^2^ test and P<0.05 considered as inequivalent. Publication bias was evaluated by depicting funnel plots and quantified by Egger's test. Data analyses were carried out using RevMan 5.3 and STATA12.0.

## Results

### Baseline characteristics of eligible literatures

Initially, 157 literatures in PubMed, 151 in Web of Science, 1 in CNKI, 77 in VIP and 15 in Wanfang database were searched out, with a total of 395 literatures. A total of 62 replicates and 287 irrelevant literatures were excluded after the first-round screening. Subsequently, 14 literatures on mechanisms, 6 reviews, 6 literatures reporting other diseases, 2 literatures without complete data and 2 reporting other mutant sites were excluded. Finally, 16 literatures were included in this study (Figure 1).

Baseline characteristics of eligible literatures were listed in Table 1. Briefly, 16 case-control studies were published from 2004-2018, including 13 studies published in English-language scientific journals and 3 in Chinese-language scientific journals. Genotyping methods were conducted using polymerase chain reaction (PCR), PCR-RFLP and PCR-sequence. Identification of single nucleotide polymorphisms (SNPs) was conducted by extracting blood samples of subjects.

**Table 1:** Main characteristics of studies included in the meta-analysis. SNP=Single nucleotide polymorphism; HWE = Hardy-Weinberg equilibrium; pHWE=p-value of Hardy-Weinberg Equilibrium test in controls for each locus; PCR = polymerase chain reaction

Author	Year	Country	Journal name/<br>publication<br>origin	Genotyping<br>methods	SNP loci (P_HWE_)	Sample size	Control	Sample
Zhou	2004	China	Chinese Medical<br>Journal	PCR-<br>sequence	rs3918242<br>(pHWE=0.92)	100 (male=98,<br>female=)	100 (male=99,<br>female=1)	Whole<br>blood
Isao Ito	2005	Japan	Am J Respir Crit<br>Care Med	PCR-RFLP	rs3918242<br>(pHWE=0.41)	84 (male=81,<br>female=3)	85 (male=69,<br>female=16)	
Zhang<br>Rongbao	2005	China	Chin J Epidemiol	PCR-RFLP	rs3918242<br>(pHWE=0.09)	147 (male=135,<br>female=12)	120<br>(male=110,<br>female=10)	Whole<br>blood
Han	2006	Asian	Chin J Tuberc<br>Respir Dis	PCR-RFLP	rs3918242<br>(pHWE=0.48)	60	52	Whole<br>blood
Testaigzi	2006	Caucasian	Int J Chron<br>Obstruct Pulmon<br>Dis	PCR-RFLP	rs3918242<br>(pHWE=0.39)	123	262	Whole<br>blood
Korytina	2008	Russia	Russian Journal<br>of Genetics	PCR-RFLP	rs3918242<br>(pHWE=0.53)	318	319	Whole<br>blood
Shih-Lung<br>Cheng	2009	Taiwan<br>(China)	Biochem Genet	PCR-RFLP	rs3918242<br>(pHWE=0.23)	184 (male=152,<br>female=32)	212<br>(male=182,<br>female=30)	Whole<br>blood
H. Schirmer	2009	Brazil	Genetics and<br>Molecular<br>Research	PCR	rs3918242<br>(pHWE=0.60)	89	97	Whole<br>blood
Shih-Yup<br>Lee	2010	Korean	Basic Science<br>Investigations	PCR-sequence	rs3918242<br>(pHWE=0.376)	301	333	Whole<br>blood
Hua	2010	China	Int J Respi	PCR-RFLP	rs3918242<br>(pHWE=0.04)	180 (male=142,<br>female=38)	180<br>(male=130,<br>female=50)	Whole<br>blood
Korytina	2012	Russia	Molecular<br>Biology	PCR-RFLP	rs3918242<br>(pHWE=0.67)	391	514	Whole<br>blood
Sarra Bchir	2015	Tunisia	Mol Diagn Ther	PCR-RFLP	rs3918242<br>(pHWE=0.02)	138 (male=122,<br>female=16)	216<br>(male=155,<br>female=61)	Whole<br>blood
Marja<br>Stankovic	2016	Serbia	Environmentaland<br>Molecular<br>Mutagenesis.<br>PCR-RFLP	rs3918242<br>(pHWE=0.28)	86	100		Whole<br>blood
Marja<br>Stankovic	2017	Serbia	JOURNAL<br>OF CHRONIC<br>OBSTRUCTIVE<br>PULMONARY<br>DISEASE	PCR-RFLP	rs3918242<br>(pHWE=0.28)	122	100	Whole<br>blood
Tan Jie	2017	China	Journal<br>Of Inner<br>Mongolia<br>Medical Universit	PCR-RFLP	rs3918242<br>(pHWE<0.001)	186 (male=92,<br>female=294)	219<br>(male=105,<br>female=112)	Whole<br>blood
Lwona<br>Gilowska	2018	Poland	BioMed Research<br>International	PCR-RFLP	rs3918242<br>(pHWE=0.33)	335<br>(male=87,<br>female=248)	309<br>(male=229,<br>female=80)	Whole<br>blood

In the 16 eligible literatures, 5 analyzed Chinese population, 1 analyzed Japanese population, 2 analyzed Russian population, 1 analyzed Brazilian population, 1 analyzed Korean population, 1 analyzed Tunisian population, 2 analyzed Serbian population, 1 analyzed Poland population, 1 analyzed Asian population and 1 analyzed Caucasian population. Sample size of each literature was 60-391.

### Meta-analysis

**Figure 2:**
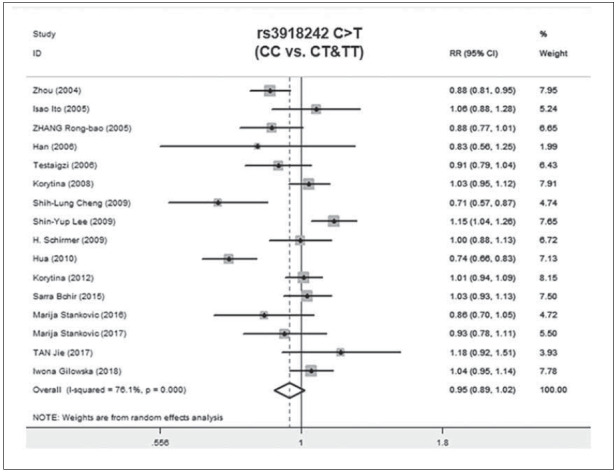
Forest map of the relationship between the SNP of MMP-9 rs3918242 and susceptibility to COPD

**Figure 3:**
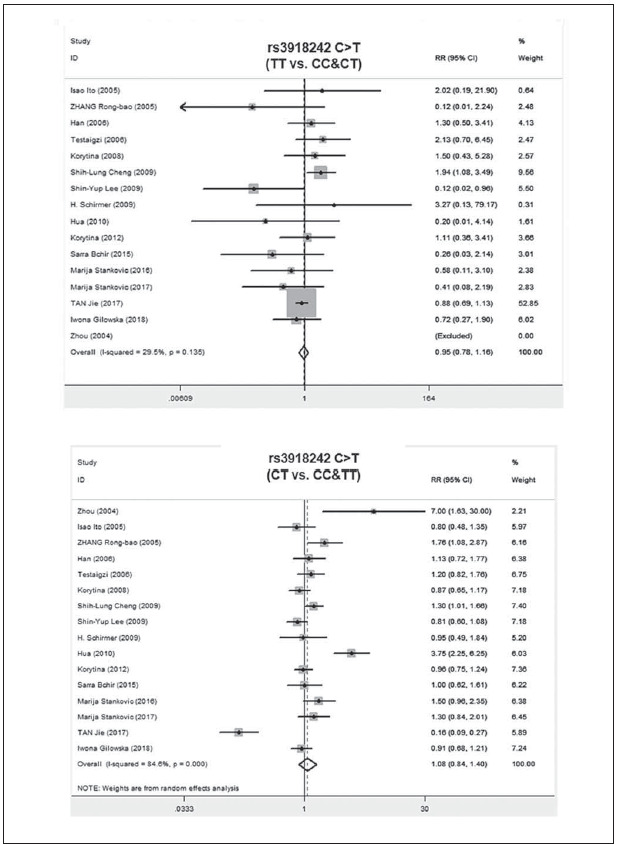
Forest map of the relationship between the SNP of MMP-9 rs3918242 and susceptibility to COPD

**Figure 4:**
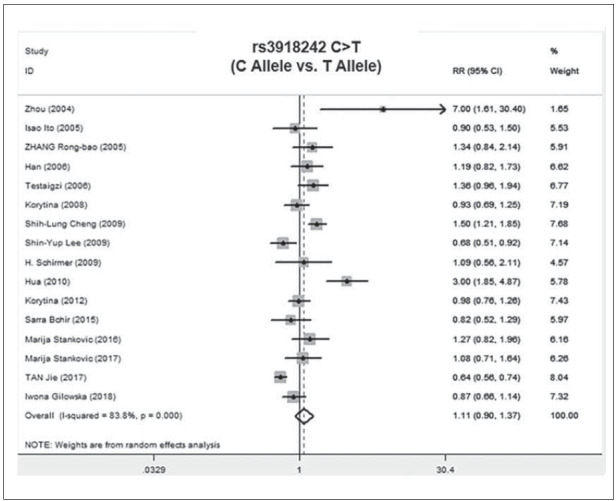
Forest map of the relationship between the SNP of MMP-9 rs3918242 and susceptibility to COPD

**Figure 5:**
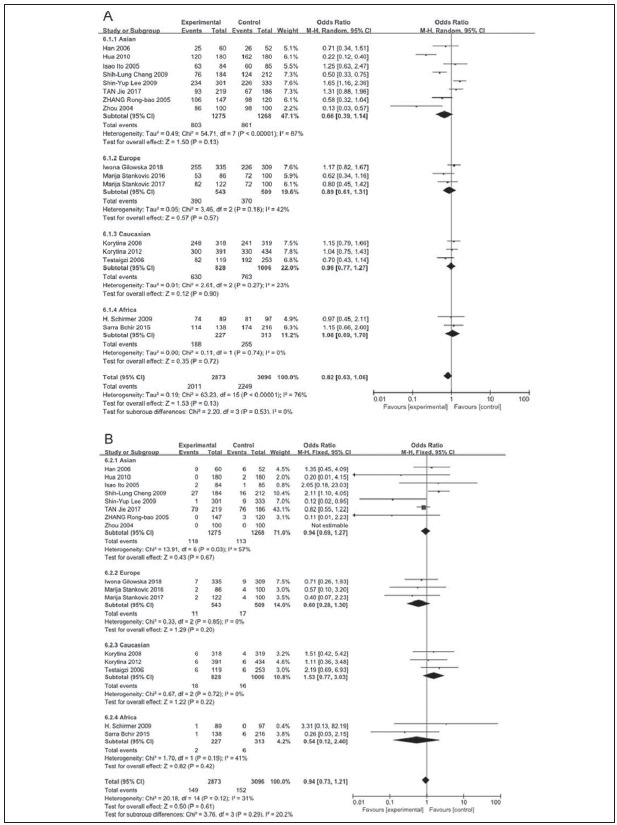
Subgroup analyses of the relationship between the SNP of MMP-9 rs3918242 and susceptibility to COPD in different regions and different pairs of comparisons

A total of 2011 COPD patients and 2249 healthy controls were enrolled. The influence of MMP9 (-1562) C/T on susceptibility to COPD was assessed using different genetic models. No relationship was found between the CC vs.TT genotype of MMP9 rs391842 and susceptibility to COPD in the allele model (P=0.41, OR=1.12, 95% CI=0.86-1.47) (Figure 2, Figure 3 and Figure 4). The other three genetic models obtained the same conclusion, including the dominant model (CC vs. CT+TT, P=0.13, OR=0.82, 95% CI=0.63-1.06), recessive model (TT vs. CC+CT, P=0.87, OR=0.97, 95% CI=0.65-1.43) and over-dominant model (CT vs. CC+TT, P=0.51, OR=1.13, 95% CI=0.79-1.61). Figure 5

**Figure 6:**
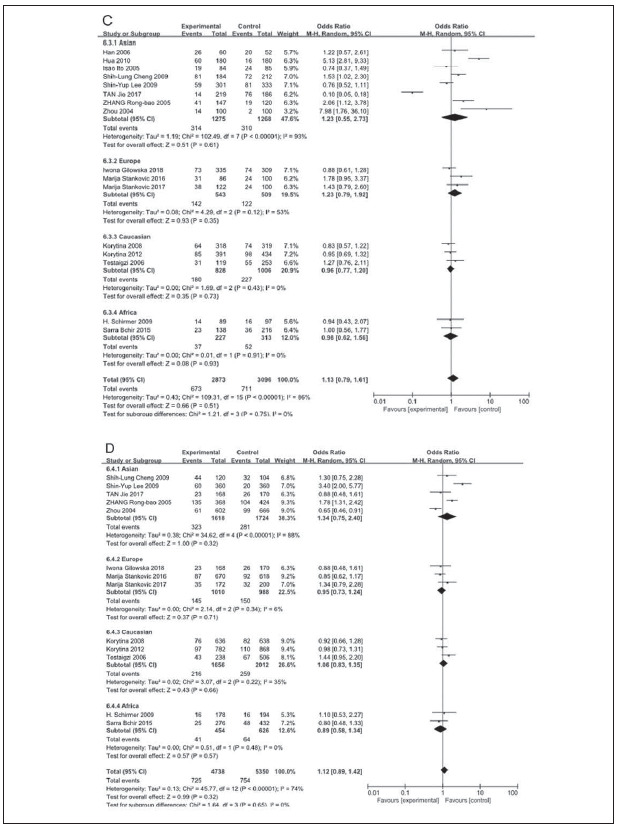
Subgroup analyses of the relationship between the SNP of MMP-9 rs3918242 and susceptibility to COPD in different regions and different pairs of comparisons

Subgroup analyses were performed based on the ethnic populations, involving Asian population (8 literatures), European population (3 literatures), Caucasian population (3 literatures) and African population (2 literatures). The random-effects model was utilized owing to the different degrees of heterogeneity (I^2^ >50%, P<0.05). The data showed no relationship between MMP9 polymorphisms and COPD risk under the different genetic models (P>0.05) (Figure 6).

**Figure 7:**
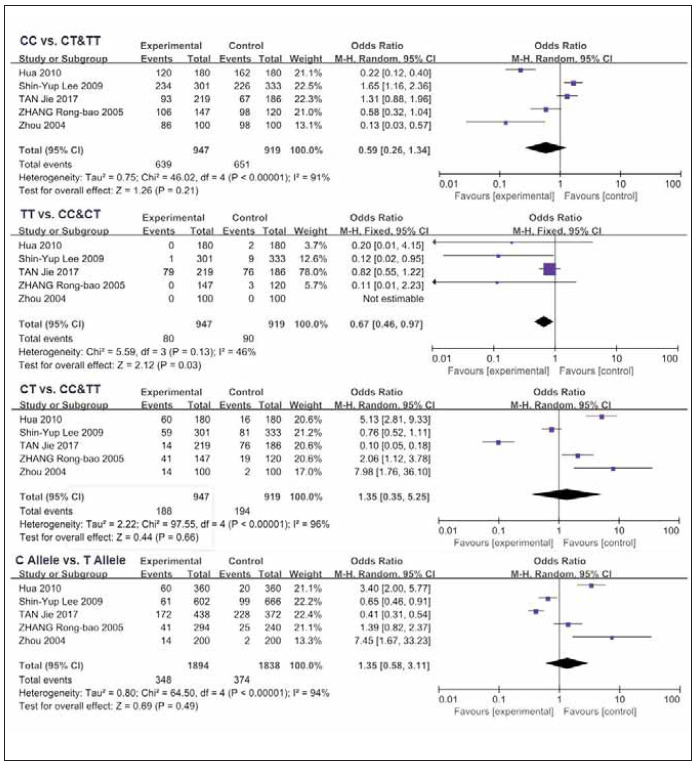
Subgroup analyses of the relationship between the SNP of MMP-9 rs3918242 and susceptibility to COPD in Chinese population and different pairs of comparisons

Subsequently, we individually analyzed the relationship between MMP9 polymorphisms and COPD in Chinese population, involving 5 literatures [15] [18] [21] [22] [23]. Except for the recessive model (TT vs. CC&CT) analyzed by the fix-effects model (P=0.13, *I^2^* =46%), the remaining were assessed using the random-effects model (*I^2^* >50%, P<0.05) (Figure 7). Our data showed that Chinese population carrying the TT genotype for the MMP-9 rs3918242 was closely related to susceptibility to COPD relative to those carrying CT and CC genotypes (P=0.03, OR=0.67, 95% CI=0.46-0.97). Such a difference was not observed in the dominant model (CC vs. CT&TT), over-dominant model (CT vs. CC&TT) and allele model (C Allele vs. T Allele) (P>0.05) (Figure 7).

### Heterogeneity and sensitivity analysis

Significant heterogeneity was identified in the dominant model, over-dominant model and allele model analyzing the relationship between MMP9 (-1562) C/T and susceptibility to COPD (all P<0.001). No remarkable changes in I^2^ and P values were observed after removing a single study. In addition, sensitivity analysis was not altered by removing any study each time (data not shown).

In the subgroup analyses based on different ethnic populations, all genetic models showed the results of I^2^ >50% and P<0.05. We did not find any changes in I^2^ and P values after removing a single study. Sensitivity analysis was not influenced by removing a single study (data not shown).

### Publication bias

**Figure 8:**
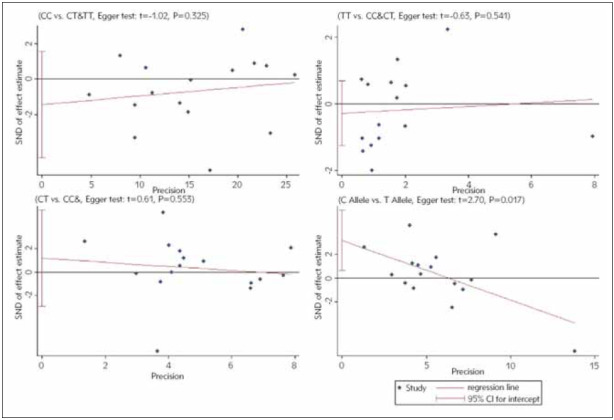
Subgroup analyses of the relationship between the SNP of MMP-9 rs3918242 and susceptibility to COPD in Chinese population and different pairs of comparisons

A wide range of search strategies was carried out to minimize potential publication biases. After quantification using Egger's test, the data showed no publication biases between MMP9 (-1562) C/T and susceptibility to COPD in the three genetic models except for the allele model (CC vs. CT+TT, P=0.325; TT vs. CC+CT, P=0.541; CT vs. CC&TT, P=0.553; C allele vs. T allele, P=0.017) (Figure 8).

## Discussion

MMPs are a class of zinc-dependent endopeptidases that degrade major protein components of the ECM. They participate in development- and inflammation-related tissue remodeling and repair [7]. MMP-9 (gelatinase B) can degrade ECM proteins, such as type IV collagen and gelatin [24]. In addition, it exerts a vital role in airway inflammation and remodeling [25] [26]. MMP-9 protects ventilatorinduced lung injury by reducing infiltration of alveolar neutrophils [27].

COPD is a common respiratory disease characterized by airflow limitation. The pathogenesis of COPD is complex, involving inflammatory response, oxidant-antioxidant imbalance, and MMPs-induced proteolysis of the alveolar wall. MMP9, one of the most widely studied MMPs, decomposes most of the components of ECM by degrading structural proteins, such as collagen and elastin [28]. Many studies have reported the involvement of MMP9 in the development of lung diseases [29]. MMP9 polymorphism is identified to increase the susceptibility to respiratory diseases [30] [31] [32] [33]. Multiple SNPs of MMP9 have been discovered. Among them, C/T mutation on MMP9 (-1562) rs3918242 results in the increased promoter activity owing to the deletion of the transcriptional repressor binding site [34].

So far, studies focusing on the correlation between MMP9 -1562 C/T polymorphism and COPD are relatively rare and uncertain. Studies with a small sample size lack the statistical power and often lead to contradictory conclusions. Meta-analysis provides convincing evidences by calculating data extracted from multiple studies. In this paper, we obtained the conclusion that MMP9 -1562 C/T polymorphism was not associated with susceptibility toped in different putative genetic models. Subgroup analyses showed that Chinese population carrying the TT genotype for the MMP-9 rs3918242 are risky of COPD relative to those carrying CT and CC genotypes.

Inconsistent with our results, some studies have demonstrated that the MMP9 -1562 C>T polymorphism indeed influences COPD risk. Zhou et al. [35] illustrated that the TT genotype of MMP9 -1562 C/T polymorphism is a genetic risk factor for severe COPD. Korytina et al. [36] have indicated the correlation between the TT genotype of MMP9 -1562 C/T polymorphism and COPD severity. Similarly, a study conducted in Russia showed a significant difference in the frequency distribution of MMP9 -1562 C>T among COPD patients with different severity levels [37].

Some shortcomings in this study should be pointed out. First of all, many complex factors were not adjusted, such as gender, age, and smoking history. Secondly, some studies [16] [20] [23] had small sample sizes and did not have enough capacity to detect the risk of COPD. Thirdly, the lack of raw data limited the further analysis of the potential interactions between genetic risks and environmental factors in COPD. Studies with large sample sizes in a multicenter hospital are required for further validation.

## Conclusions

Chinese population carrying the TT genotype for the MMP-9 rs3918242 present lower susceptibility to COPD relative to those carrying CT and CC genotypes.

## Dodatak

### Acknowledgements

No.

### Financial Disclosure

This study was supported by the Key Project of Qingdao 2020 Traditional Chinese Medicine Scientific Research Plan (No: 2020-zyz002) and Shandong Traditional Chinese Medicine Science and Technology Project (No: 2020M108).

### Conflict of interest statement

All the authors declare that they have no conflict of interest in this work.
